# Food-Derived Multi-Target Antihypertensive Peptides: Sources, Mechanisms and AI-Driven Strategies

**DOI:** 10.3390/foods15132349

**Published:** 2026-07-02

**Authors:** Miao Zhang, Haiyang Liu, Yinuo Wang, Guodong Yu, Mengyao Liu, Zhichao Lu, Fengjiao Mao, Zhen Wu, Daodong Pan, Maolin Tu

**Affiliations:** 1 State Key Laboratory for Quality and Safety of Agro-Products, Zhejiang Key Laboratory of Food Microbiology and Nutritional Health, Zhejiang-Malaysia Joint Research Laboratory for Agricultural Product Processing and Nutrition, College of Food Science and Engineering, Ningbo University, Ningbo 315211, China; 19860917176@163.com (M.Z.); liuhaiyang11242025@163.com (H.L.); wangyinuo070919@163.com (Y.W.); 2411390099@nbu.edu.cn (G.Y.); wuzhen@nbu.edu.cn (Z.W.); daodongpan@163.com (D.P.); 2 Key Laboratory of Geriatric Nutrition and Health, Beijing Technology and Business University, Beijing 100048, China; 20230200@btbu.edu.cn; 3 School of Biological and Materials Engineering, Suqian University, Suqian 223800, China; luzhichao1s@163.com

**Keywords:** hypertension, multi-target antihypertensive peptides, artificial intelligence, network pharmacology, molecular docking

## Abstract

Hypertension is a major global public health challenge. Traditional antihypertensive drugs often cause side effects, which has prompted growing interest in natural antihypertensive agents. However, most existing antihypertensive peptides target single pathways, thereby constraining their effectiveness against hypertension’s complex mechanisms. In contrast, multi-target peptides modulate complex hypertension-related networks, offering enhanced blood pressure control and reduced resistance risks. This narrative review comprehensively summarizes the latest research progress on multi-target antihypertensive peptides, including their main food sources (animal, plant, and microorganism sources) and bioactive mechanisms. In addition, this review also describes the process of artificial intelligence (AI) and network pharmacology-driven multi-target antihypertensive peptide screening, and summarizes the machine learning (ML) models and activity prediction websites that have been applied to antihypertensive peptide screening. Finally, this review explores the challenges and future directions in multi-target antihypertensive peptide research, thereby providing a theoretical basis for the development of novel multi-target antihypertensive peptides.

## 1. Introduction

Hypertension is one of the most important and preventable risk factors for cardiovascular disease, and its global prevalence continues to rise, making it a major challenge in public health. Many types of drugs have been developed for the treatment of hypertension, such as diuretics, beta-blockers, calcium channel blockers, angiotensin-converting enzyme inhibitors, and angiotensin II (Ang II) receptor blockers, among others [1]. However, these drugs are not only expensive but also tend to cause various side effects, such as dry cough, headache, dry mouth, insomnia, and depression in hypertensive patients [2,3,4].

In recent years, with concerns about the safety of chemically synthesized drugs, there has been an increasing focus on obtaining healthy ingredients with antihypertensive activity from natural sources [5]. Some studies suggest that antihypertensive peptides derived from a variety of food sources offer better advantages than existing drugs, such as fewer side effects and less toxicity, among other benefits [6]. Antihypertensive peptides have been extracted from a variety of foods, such as milk, black soybean, chicken, algae, *Chlamydomonas reinhardtii,* and corn [7,8,9]. Most of the current antihypertensive peptides are single-target, but the blood pressure-lowering mechanism is extremely complex, and the single-targeted mechanism of action makes it difficult to comprehensively regulate the complex blood pressure regulatory network (e.g., the renin-angiotensin system, the endothelin pathway, among others), resulting in insufficient efficacy in some patients [10]. Consequently, multi-target synergistic therapy has become a new direction in the development of hypertension drugs, and bioactive peptides of natural sources for lowering blood pressure have gradually become the focus of research due to their multiple regulatory potential, high safety profile and food compatibility. Within the scope of this review, multitarget antihypertensive peptides are defined as bioactive peptides capable of simultaneously acting on two or more key targets (e.g., enzymes, receptors, or signaling pathways) within the blood pressure regulatory network. They achieve antihypertensive effects through the synergistic modulation of multiple physiological mechanisms. Certain antihypertensive peptides have been shown to simultaneously inhibit angiotensin-converting enzyme (ACE), renin, and endothelin-converting enzyme (ECE), while upregulating angiotensin converting enzyme 2 (ACE2) expression and enhancing nitric oxide (NO) release. For example, Ma et al. [11] extracted peptides LGF and GLFF from *Moringa oleifera*, which have dual inhibitory activities on renin and ACE, and demonstrated in vivo that two peptides can significantly reduce systolic and diastolic blood pressure in spontaneously hypertensive rats (SHRs). Although numerous studies have successfully extracted antihypertensive peptides from natural food sources, traditional experimental screening methods (e.g., directed enzymatic hydrolysis and activity validation) still dominate. Traditional experimental approaches often suffer from inefficiency and high costs, severely limiting the large-scale discovery and application of antihypertensive peptides [12].

The rise of AI technology offers new ways to address bottlenecks in traditional antihypertensive peptides screening. By integrating multidimensional bioinformatics data, such as sequence features, spatial conformation, and target interaction networks, AI can construct an efficient computational model. The AI-based approach significantly enhances the screening efficiency of bioactive peptides, shortens the screening cycle, and reduces costs [13]. With the development of computer technologies like machine learning (ML) and neural networks, functional characterization of peptides has become possible [14]. Various ML models, such as support vector machine (SVM), random forest (RF), convolutional neural networks (CNN), graph neural networks (GNN), have been used to screen antihypertensive peptides [15,16]. For instance, Zhang et al. [17] assessed the performance of four deep learning (DL) models, BERT_base, ProtBERT, LSTM (long short term memory) and CRNN (convolutional recurrent neural network), and ultimately demonstrated that the ProtBERT model performed the best, with an AUC value of 0.9785, an accuracy of 0.93, and a recall, specificity, precision, and F1 score of 0.93 or more. Using ProtBERT predictions and molecular docking, the study identified three antihypertensive peptides: LVPFGW, VSFPVL, and VLPF. Later experiments confirmed that these peptides had strong activity in vitro. Similarly, Damen et al. [18] successfully screened and identified three antihypertensive peptides (LRF, GAWY, and QW) with significant ACE inhibitory activity from protein hydrolysates derived from dairy white wastewater containing milk proteins, by integrating peptidomics analysis with ML prediction models and validating their findings through in vitro experiments. These examples illustrate the potential of AI to identify novel antihypertensive peptides. Although AI has already been used for the screening of antihypertensive peptides, currently, AI faces difficulties in directly predicting multi-target antihypertensive peptides. It is worth noting that network pharmacology has developed into an effective approach for dissecting multi-target mechanisms of action in complex diseases [19]. Therefore, based on existing studies, the integration of artificial intelligence, network pharmacology, and molecular docking may offer an efficient and cost-effective approach for the screening and prediction of food-derived peptides with multi-target antihypertensive activities, while also facilitating the elucidation of their mechanisms. However, there remains a critical void in specialized reviews that comprehensively integrate the food sources, mechanisms of action, and the application of AI in the discovery of these multi-target antihypertensive peptides. Therefore, this review aims to bridge this crucial gap by providing a comprehensive analysis of the sources and bioactive mechanisms of multi-target antihypertensive peptides, critically examining the role and inherent challenges of AI techniques in their screening, and outlining future research directions.

This review systematically searched the Web of Science and PubMed databases for literature published between 2010 and 2026. The search keywords included: “food-derived antihypertensive peptides”, “multi-target antihypertensive peptides”, “ECE”,”ACE2”, “ACE”, “renin”, “eNOS”, “machine learning”, “deep learning”, “AI”, and “network pharmacology”. Inclusion criteria comprised studies explicitly investigating multi-target/multi-pathway mechanisms or utilizing AI for antihypertensive peptide screening. Exclusion criteria covered studies only reporting single-target antihypertensive peptides without employing AI for screening.

## 2. Sources of Multi-Target Antihypertensive Peptides

Research on food-derived multi-target peptides with blood pressure-lowering effects has diversified significantly in recent years. These peptides have been developed from a wide variety of sources, including microorganisms, plant proteins, and animal proteins. These peptides have exhibited considerable potential for bioactivity and industrial applications.

### 2.1. Plant-Derived Multi-Target Antihypertensive Peptides

Some multi-target antihypertensive peptides were identified from various plants, including pumpkin seeds, rapeseeds, naked oats, and walnuts. For example, two novel peptides, SNHANQLDFHP and PVQVLASAYR, isolated from pumpkin seed meal (protein content: 80.87%) hydrolysate by Li et al. [20] demonstrated significant ACE inhibitory activity. Cellular experimental studies revealed that these two peptides may exert antihypertensive effects through a triple mechanism: directly inhibiting ACE activity and blocking Ang II production; subtly and bidirectionally regulating vasoactive substances, inhibiting endothelin-1 (ET-1) production; and simultaneously promoting NO release; positively upregulating ACE2 activity, providing multi-target intervention in the renin-angiotensin-aldosterone system (RAAS) and effective blood pressure regulation. Similarly, He et al. [21] isolated three bioactive peptides, LY, RALP, and GHS (derived from cruciferin), from rapeseed protein hydrolysate, and the in vitro studies showed their dual inhibitory effects on ACE and renin. Animal studies further revealed that these peptides not only inhibited ACE/renin activity but also upregulated ACE2 expression, promoted Ang-(1–7) production, and increased Mas receptor (MasR) expression [22]. This simultaneous inhibition of the classical ACE/renin axis and activation of the protective ACE2/Ang-(1–7)/Mas axis lays the groundwork for subsequent research into multi-target antihypertensive peptides. The naked oat globulin-derived (globulin accounts for 50–80% of the total protein in oat) ACE inhibitory peptide SSYYPFK exhibits promising ACE inhibitory activity and in vitro digestive stability [23]. Its mechanism for lowering blood pressure involves the inhibition of ACE and renin, along with reduced ET-1 levels. Furthermore, animal experiments by Tian et al. [24] demonstrated that lotus seed protein hydrolysates effectively reduced blood pressure in SHRs by upregulating ACE2 enzyme activity and simultaneously inhibiting ACE enzyme activity.

In addition to the aforementioned in vitro and animal experimental methodologies on screening multi-target antihypertensive peptides, the application of emerging technological tools such as network pharmacology, has provided novel methodologies for the discovery of plant-derived multi-target antihypertensive peptides. Notably, Song et al. [25] identified three multi-target antihypertensive peptides (LLPSF, LPQFF, NLPLPF) from walnut protein. Network pharmacology studies revealed renin as their core target, and these peptides achieve their blood pressure-lowering effects through synergistic interactions with multiple hypertension-related targets. This research provides a new theoretical basis for the development of plant-derived antihypertensive peptides. Similarly, Song et al. [26] employed network pharmacology analysis to identify ACE and ACE2 as core targets for the hazelnut peptide YYLLVR. Molecular docking studies subsequently confirmed its robust and stable interaction with several critical targets, specifically ACE, AGTR1, ACE2, MAS and AGTR2. Cellular experiments have confirmed that YYLLVR inhibits the release of inflammatory factors and up-regulates synapse-related proteins by acting on these targets, thereby exerting neuroprotective effects and ultimately lowering blood pressure. In addition to the above multi-target antihypertensive peptides from plants, researchers have also successfully screened multi-target antihypertensive peptides from wheat bran [27], *Sargassum maclurei* [28], broccoli [29], amaranth [30] and quinoa [31].

These findings suggest that plant-derived peptides have great potential for lowering blood pressure. Their multi-target mechanism of action may lead to improved efficacy and reduced side effects in clinical applications. In the future, studies will investigate the bioavailability and in vivo mechanism of action of these peptides, as well as the development of appropriate foods or drugs.

### 2.2. Animal-Derived Multi-Target Antihypertensive Peptides

Analogous to multi-target antihypertensive peptides from plant sources, peptides derived from animal proteins also demonstrate significant progress in recent years. Yu et al. [32] initially isolated a peptide NCW (derived from myosin) from *Mizuhopecten yessoensis*, which exhibited potent antihypertensive activity in vitro and in vivo. Subsequently, Xue et al. [33] integrated network pharmacology and molecular docking to analyze the multi-target antihypertensive mechanism of the ACE inhibitory peptide NCW from a systems biology perspective. They found that the peptide could specifically bind to 11 core targets, including ALB and MMP9, suggesting that the peptide NCW could achieve the synergistic antihypertensive effect of multiple targets through the regulation of multiple signalling pathways, such as those related to relaxin and MAPK. This study provides a theoretical basis for an in-depth analysis of the antihypertensive mechanism of peptide NCW. It is also worth noting that Wang et al. [34] successfully screened a novel ACE inhibitory peptide (LTFSY) from yellow tuna, which exhibited significant ACE inhibitory activity with an IC_50_ of 20.72 µg/mL. Additionally, network pharmacology study results showed that LTFSY may regulate blood pressure homeostasis. This regulation might occur through multi-target synergistic effects. Its core targets included key signalling molecules such as AGTR1, MAPK8, NOS3, and RHOA. The above studies predicted the potential targets of antihypertensive peptides by network pharmacology and molecular docking methods, but a critical limitation is the absence of rigorous in vivo and in vitro experimental validation, leaving their conclusions largely speculative. In order to further elucidate the antihypertensive mechanisms of multi-target peptides, future studies must continue to validate the corresponding targets in vivo and in vitro.

In addition to multi-target antihypertensive peptides prepared from marine animal sources, some studies have also prepared multi-target antihypertensive peptides from terrestrial animal sources. For example, Cao et al. [35] investigated the in vivo antihypertensive effects of bovine bone gelatin-derived peptides (derived from collagen) in SHRs. Mechanistic studies revealed that bovine bone gelatin-derived peptides exerted multi-target antihypertensive effects by downregulating the ACE/Ang II/angiotensin II type 1 receptor (AT1R) pathway and activating the Ang II/angiotensin II type 2 receptor (AT2R) pathway. The antihypertensive mechanisms of the milk-derived tripeptides VPP and IPP (derived from casein) have progressively evolved from single-target inhibition to multi-pathway synergistic actions. Early studies established that these two peptides exert their antihypertensive effects through direct inhibition of ACE activity. Subsequent investigations have demonstrated that they also lower blood pressure in both animal models and human subjects via improving endothelial function, activating ACE2, and attenuating inflammatory responses [36,37]. Possessing both multi-target synergistic effects and clinical validation, these antihypertensive peptides has laid the groundwork for further research in the field, offering a crucial reference, especially for those antihypertensive peptides lacking clinical data.

In addition to the aforementioned, studies have demonstrated the potential of *cicada chrysalis* [38], tilapia [39] as animal sources of multi-target antihypertensive peptides. In summary, some multiple antihypertensive peptides have been successfully isolated from animal proteins. Through a synergistic mechanism involving multiple pathways and targets, these peptides achieve comprehensive regulation of blood pressure. These findings provide a theoretical foundation for the development of novel antihypertensive functional foods and pharmaceuticals.

### 2.3. Microorganisms-Derived Multi-Target Antihypertensive Peptides

Some researchers have also screened multi-target antihypertensive peptides from microorganisms. For example, Zheng et al. [40] identified two antihypertensive peptides, IQP and VEP, from *Spirulina platensis*, which exhibited synergistic blood pressure-lowering effects in SHRs through dual regulatory mechanisms. These peptides simultaneously inhibited the vasoconstrictive ACE/AngII/AT1R axis and activated the vasodilatory ACE2-Ang (1–7)-Mas axis, leading to significant blood pressure reduction. Chen et al. [41] isolated the peptide FEIHCC from *Isochrysis zhanjiangensis* and confirmed its antihypertensive properties. In vitro experiments showed that the peptide had a significant inhibitory effect on ACE. Further analysis revealed that FEIHCC effectively inhibited Ang II-induced inflammatory response and apoptosis in a vascular endothelial cell model. Its active mechanisms may be related to the modulation of multiple signalling pathways such as NF-κB, Nrf2, MAPKs and Akt. These results suggest that FEIHCC may exert blood pressure-lowering effects through multiple mechanisms.

Notably, in addition to algae, researchers have also identified peptides with multi-target antihypertensive potential from fungi. Li et al. [42] initially isolated the ACE inhibitory peptide KYPHVF from the edible symbiont *Boletus griseus*-*Hypomyces chrysospermus*. Subsequent studies demonstrated that KYPHVF maintained stability during simulated digestion. Furthermore, intragastric administration of KYPHVF significantly reduced the blood pressure of SHRs within 4 h post-treatment [43]. Network pharmacology combined with molecular docking demonstrated its multi-target antihypertensive effects through 11 targets including MAPK14 and MAP2K1. In vivo studies conducted by Huan et al. [44] further confirmed the multi-target hypotensive potential of KYPHVF. The peptide KYPHVF achieves synergistic blood pressure reduction by inhibiting the vasoconstrictive ACE/Ang II/AT1R axis, activating the vasodilatory ACE2-Ang(1–7)-MAS1 pathway, and modulating the gut microbiota.

In recent years, there has been an increasing focus on the study of natural bioactive peptides, especially those from microorganisms. In comparison with other sources, microorganism-derived peptides may possess certain advantages, including improved sustainability and higher cost-effectiveness [45,46], which could enhance their commercial viability.

In summary, food-derived multi-target antihypertensive peptides from plants, animals, and microorganisms have been extensively identified. However, the level of evidence supporting the multi-target activity of these peptides varies considerably across studies. Among the aforementioned multi-target antihypertensive peptides, peptides such as LY, RALP, GHS, IQP, VEP, and KYPHVF have been rigorously evaluated through long-term studies in SHRs. However, a large number of reported peptides remain at a preliminary stage, with their multi-target effects primarily deduced from in vitro assays or computational models, lacking sufficient in vivo validation. Moreover, current research still presents several uncertainties and limitations, with most studies lacking a comprehensive evaluation of the gastrointestinal stability and bioavailability of these multi-target antihypertensive peptides. Overall, current research on multi-target antihypertensive peptides is largely at an early phase, with a general paucity of human clinical trial data and systematic evaluation of in vivo bioavailability. Notably, the milk-derived tripeptides VPP and IPP represent thoroughly investigated exemplary peptides. Beyond extensive in vitro and in vivo validation, these two peptides possess the robust body of evidence to date, with both their efficacy and safety having been substantiated through human clinical trials.

## 3. Functional Mechanisms of Multi-Target Antihypertensive Peptides

**Figure 1:**
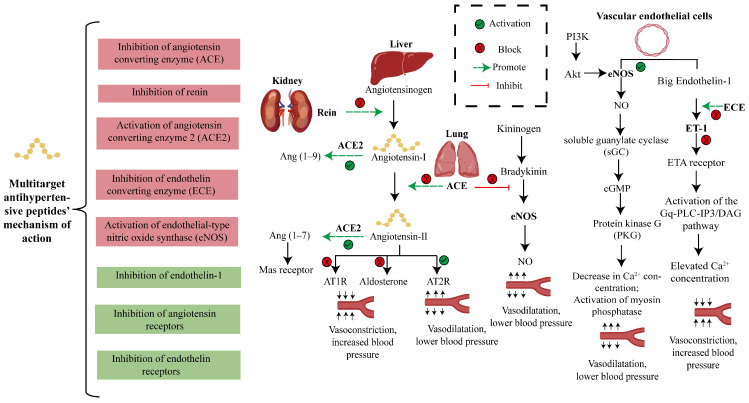
Mechanism of multi-target antihypertensive peptides.

Multi-target antihypertensive peptides lower blood pressure by acting on multiple targets, with their functional mechanisms primarily manifested in the synergistic regulation of several signaling pathways. These include the RAAS [15], the endothelial nitric oxide synthase (eNOS)-mediated NO generation system, and the endothelin system (ETS) [47]. These peptides exert their antihypertensive effects through multiple synergistic mechanisms, including influencing the activity of substances such as renin, inhibiting the activity of enzymes, and improving vascular endothelial function. Figure 1 primarily illustrates the signaling pathways and molecular targets involved in the action mechanisms of multi-target antihypertensive peptides.

### 3.1. Co-Regulation of RAAS-Related Targets

The RAAS, serving as a central pathway for the regulation of blood pressure, holds a core position in the realm of antihypertensive peptide research. Currently, substantial and comprehensive in vitro and in vivo experimental evidence has established the effective modulatory actions of these peptides on the RAAS network. The key targets of this system are known to include ACE, ACE2, renin, AT1R, and AT2R, among others [15]. Among these targets, ACE is the most extensively studied and robustly evidenced core target.

Renin and ACE are the central regulators of the RAAS pathway [48], which begins with renin catalyzing the hydrolysis of angiotensinogen to produce the decapeptide angiotensin I (Ang I), followed by ACE cleaving the two amino acid residues at the C-terminal end of Ang I to convert it to the octapeptide Ang II, which has potent vasoreactivity. Ang II is involved in blood pressure regulation by a dual mechanism: When bound to the AT1R, Ang II induces vascular smooth muscle cell (VSMC) contraction and stimulates aldosterone secretion, leading to renal sodium and water retention and ultimately an increase in blood pressure [49]. In contrast, Ang II binding to the AT2R activates vasodilator pathways, promotes NO release and inhibits cell proliferation [15]. On the other hand, ACE is the main enzyme that degrades bradykinin. Bradykinin activates endothelial cells to release nitric oxide and prostaglandins, causing vasodilation and lowering blood pressure. Therefore, ACE reduces the vasodilatory effect of bradykinin by breaking it down, leading to an increase in blood pressure [4]. In 2000, ACE2 was discovered as a homolog of ACE [50] sharing 42% sequence similarity but exhibiting opposing functions, and it regulates blood pressure through a dual mechanism. First, ACE2 can catalyze the cleavage of the C-terminal peptide of angiotensin I (Ang I) to form the natriuretic peptide angiotensin 1-9 (Ang (1–9)), thereby reducing the production of Ang II. Alternatively, ACE2 can act directly on Ang II. ACE2 cleaves the C-terminal phenylalanine residue of Ang II to form Ang (1–7), which binds to the MasR, causing vasodilation and lowering blood pressure [51,52].

Recent studies have shown that food-derived antihypertensive peptides can achieve bidirectional regulation of the RAAS system through multi-target synergistic effects. Zou et al. [27] demonstrated that wheat bran peptides lowered blood pressure in SHRs by simultaneously inhibiting ACE and renin, with stronger effects observed in peptides of smaller molecular weight. These findings confirm that wheat gluten peptides could potentially have antihypertensive properties through the synergistic inhibition of both the ACE and renin. Multi-target antihypertensive peptides targeting the RAAS system exert synergistic antihypertensive effects while their pleiotropic features may trigger cross-modulation of other physiological systems, including, but not limited to, molecular targets of the endothelin pathway and inflammation-associated signaling pathways. For example, Li et al. [53] found that the peptide derived from *Mytilus edulis* had ACE-inhibitory activity and was capable of significantly lowering blood pressure in SHRs. The antihypertensive mechanism is multidimensional and synergistic, through simultaneous inhibition of the ACE/Ang II/AT1 axis and activating the ACE2/Ang-(1–7)/Mas axis, improving vascular endothelial function, inhibiting the pro-fibrotic TGF-β/Smad pathway, and regulating gut dysbiosis. This multi-target synergistic mode of action not only explains its sustained antihypertensive action, but also provides a scientific basis for its cardiovascular protective effect.

### 3.2. Co-Regulation of Targets Related to Vascular Endothelial Function

Vascular endothelial function plays a key role in the development of hypertension. Vascular endothelial cells finely regulate vasodilatory and contractile functions and maintain blood pressure homeostasis through the secretion of a variety of vasoactive substances such as NO and endothelin-1 (ET-1). Among the many endothelial regulatory systems, the nitric oxide system mediated by nitric oxide synthase (NOS) and the ETS are particularly closely related to blood pressure regulation. Among them, eNOS, ECE, and endothelin and its receptor are important molecular targets in the regulation of vascular tone and blood pressure.

NO, an important endogenous vasodilator, plays a central protective role in maintaining vascular homeostasis. Its biosynthesis is catalyzed by eNOS in endothelial cells, which produces NO by catalyzing L-arginine. NO diffusion into vascular smooth muscle cells activates soluble guanylate cyclase (sGC), which contributes to the elevation of cyclic guanosine monophosphate (cGMP) levels, which in turn reduces intracellular calcium ion concentration, ultimately leading to vasodilation [54]. In recent years, it has been found that multi-target peptides significantly enhance eNOS activity by targeting the PI3K/Akt/eNOS [55] and PPAR-γ/caspase3/MAPK/eNOS [54] pathways. ET-1 exerts an antagonistic effect on the physiological function of NO, and ET-1 is a potent vasoconstrictor [56]. The specific binding of ET-1 to endothelin receptor triggers a series of physiological and pathological responses, including vascular smooth muscle contraction, cell proliferation, and regulation of vascular tone, etc. ECE is a key enzyme in the process of ET-1 generation, which is capable of converting macromolecular endothelin precursors into ET-1. In light of the regulatory mechanisms described above, interventions targeting the ET-1 signaling pathway demonstrate significant antihypertensive effects; ECE inhibitors exert their effects by suppressing ET-1 production, while endothelin receptor antagonists function by competitively binding to the receptor and blocking signal transduction.

Multi-target antihypertensive peptides acting on targets related to vascular endothelial function have mechanisms of action that involve not only vascular endothelial function, but may also have synergistic effects by affecting signalling pathways in other systems. For example, Xiang et al. [57] systematically elucidated the antihypertensive mechanisms of the garlic-derived peptide HDCF through an integrated approach encompassing network pharmacology, transcriptomics, in vitro cellular and in vivo studies. HDCF exerts its synergistic hypotensive effects by targeting key hypertension-related targets such as renin and ACE, alongside significantly ameliorating endothelial dysfunction via the activation of the PI3K/Akt/eNOS and cGMP/PKG signaling pathways. In another study, Zheng et al. [28] successfully isolated and characterized a novel peptide RWDISQPY from the protein hydrolysate of *Sargassum maclurei*. This peptide exhibited significant ACE inhibitory activity, featuring an IC_50_ of 72.24 μM and competitive inhibition mode. Furthermore in vivo experiments verified its effect on reducing blood pressure levels in SHRs. The study further revealed that the antihypertensive effect of RWDISQPY is the result of a dual regulatory mechanism: reducing Ang II production by directly inhibiting the activity of the enzyme ACE, down-regulating the expression level of ET-1.

## 4. AI Drives Multi-Target Antihypertensive Peptides Research

**Figure 2:**
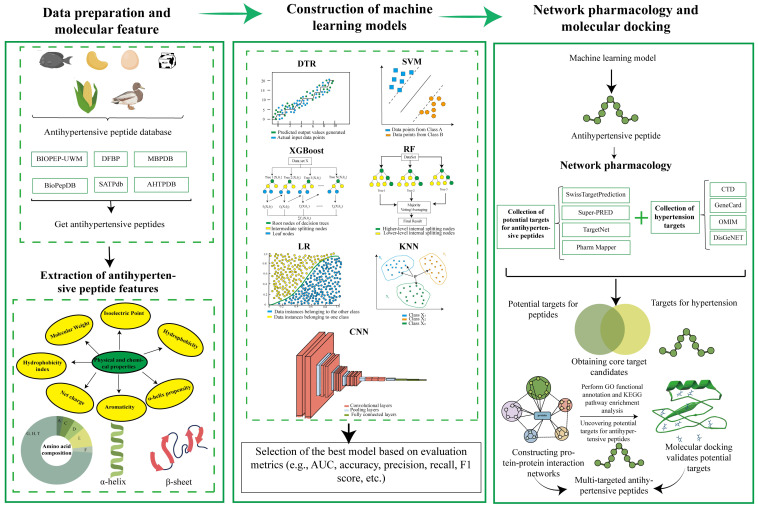
Flowchart of AI-assisted network pharmacology for screening multi-target antihypertensive peptides (The nodes in the protein-protein interaction network were divided into modules based on topological centrality, with each module represented by a distinct color).

Traditional wet-lab experiments for screening bioactive peptides are often hampered by extended experimental periods, high financial outlay, and low throughput. Such constraints significantly impede the efficient discovery of active peptides [58]. In contrast, in silico analysis offers an efficient and economical approach to accurately identify prospective bioactive peptides from vast sequence libraries, thereby drastically reducing the time and cost of the entire screening process [59]. Both conventional computational simulation methodologies, encompassing techniques such as molecular docking, quantitative structure-activity relationship (QSAR) analysis, and molecular dynamics simulations, and emergent AI approaches, critically rely on high-quality data input for their effective operation. Distinct differences exist among various in silico computational simulation methodologies. Traditional approaches, encompassing molecular docking and QSAR, are driven by biophysical and physicochemical rules. While these methods offer highly interpretable binding modes and clarified mechanisms of action as their main strengths, they are simultaneously limited by a comparatively low screening throughput and elevated computational demands. In contrast, AI enables high-throughput screening of bioactive peptides with low computational cost; however, AI models also exhibit a pronounced “black-box” nature in their internal decision-making mechanisms, resulting in limited interpretability [60,61]. Significantly, AI technologies can also function as an enhancement tool, improving the efficacy of traditional methods like molecular docking and QSAR [62,63]. In the field of antihypertensive peptide screening, both traditional computational simulations and AI-driven approaches hold broad yet distinct prospects. Conventional methods such as molecular docking and molecular dynamics will continue to play an irreplaceable role in elucidating mechanisms of action and visualizing binding modes AI methodologies, leveraging their ultra-high-throughput preliminary screening capability, their advantage in uncovering complex non-linear activity relationships, and their potential through multi-task learning to enable high-throughput identification of multi-target active peptides, are poised to play a pivotal role in the early stages. Therefore, this review recommends initially using AI for the primary screening of antihypertensive peptides, then employing network pharmacology for multi-target prediction, and finally using molecular docking for functional validation and mechanism elucidation of candidate multi-target antihypertensive peptides, achieving a closed loop of AI-driven, data- and physics-deep integration, greatly accelerating multi-target antihypertensive peptides. Figure 2 clearly illustrates the steps involved in AI-driven screening of multi-target antihypertensive peptides combined with network pharmacology approaches. The figure also lists commonly used and key databases, ML models, and related analytical tools in this research area.

### 4.1. Efficient Screening of Antihypertensive Peptides Based on ML

AI has achieved significant advancements in recent years, particularly in ML and DL [64]. AI techniques exhibit strong promise in the target prediction, activity screening, mechanism of action elucidation and structure-activity relationship studies for multi-target antihypertensive peptides, thereby providing new strategies to accelerate their screening. Mo et al. [65] used deep learning to optimize the enzymatic hydrolysis process and, through in vitro and in vivo experiments, demonstrated that walnut hydrolysate reduces blood pressure through a synergistic effect of enhancing the body’s antioxidant capacity and inhibiting ACE activity. This study significantly reduced the time and cost associated with screening for antihypertensive peptides. Therefore, the use of ML techniques to help discover and screen antihypertensive peptides is of great value. The application of ML in the screening of antihypertensive peptides usually follows the following process: First, data preparation and preprocessing. Next, transformation of the peptide sequences into computable feature vectors through molecular feature representation, then construction and training of the predictive model. Finally, ensuring the reliability of the model through rigorous evaluation and validation. This series of interrelated steps constitutes a complete intelligent screening system for antihypertensive peptides.

#### 4.1.1. Bioactive Peptide Databases and Molecular Feature

The construction and sharing of food-derived peptide databases are an important cornerstone of AI-driven multi-target antihypertensive peptides research. In ML systems, data as a fundamental element constitutes the core support for algorithm training, and its scale, quality, and diversity features are significantly positively correlated with model performance, which ultimately determines the accuracy and reliability of prediction results by affecting the feature characterization ability and generalization level [66]. With the rapid advancement of high-throughput sequencing technology and the expansion of experimental data on antihypertensive peptides in recent years, several peptide databases have been developed, boasting high-precision and multi-dimensional annotations. These databases furnish an essential standardized data basis for applying ML to antihypertensive peptides research, consequently accelerating their discovery and prediction.

Representative databases include BIOPEP-UWM (https://biochemia.uwm.edu.pl/biopep-uwm/ (accessed on 28 April 2026)) [67], BioPepDB (http://bis.zju.edu.cn/biopepdbr/index.php (accessed on 28 April 2026)) [68], SATPdb (http://crdd.osdd.net/raghava/satpdb/ (accessed on 28 April 2026)) [69], DFBP (http://www.cqudfbp.net/ (accessed on 28 April 2026)) [70], MBPDB (https://mbpdb.nws.oregonstate.edu (accessed on 28 April 2026) ) [71]. AHTPDB (http://crdd.osdd.net/raghava/ahtpdb/ (accessed on 28 April 2026)) [72] is a comprehensive database specifically designed for antihypertensive peptides, containing peptide entries and providing detailed annotation information including sequence information, IC_50_ values, toxicity/bitterness parameters, sources, molecular weights, isoelectric points and purification methods. The database also integrates predicted secondary and tertiary structure data, highly valuable for training and validating ML models.

These databases include peptide sequences, structural characterization and functional information from various sources, providing an important database for training ML models. Applying AI to screen antihypertensive peptides shows significant potential despite methodological challenges. Enhanced database curation enables more accurate resources, directly facilitating the refinement of ML models for robust peptide prediction.

#### 4.1.2. ML Models for Screening and Prediction of Antihypertensive Peptides

ML is divided into three main categories: Reinforcement learning, supervised learning and unsupervised learning. Supervised learning is an ML method where algorithms learn through labelled training data. Unsupervised learning is an ML method in which algorithms learn from unlabeled training data. Reinforcement learning is another area of ML, alongside supervised and unsupervised learning. It involves an agent interacting with an environment, in which the agent adapts its strategy based on reward signals fed back from the environment to maximize cumulative rewards in the long run [73]. Supervised learning shows greater advantages in terms of goal clarity, algorithm maturity, interpretability and data efficiency. Supervised learning models such as RF, eXtreme Gradient Boosting (XGBoost) and SVM have been widely applied to the screening of antihypertensive peptides. The machine models and their web servers used in the current study for antihypertensive peptides screening are summarized in Table 1.

Despite the extensive implementation of ML models in the screening of antihypertensive peptides, it is imperative to acknowledge the necessity of validation of the peptides identified by computational modelling. In vitro activity assays and animal model experiments are crucial for the substantiation of the peptides’ blood pressure-lowering potential and their therapeutic efficacy.

### 4.2. AI-Driven Target Identification and Pathway Analysis of Multi-Target Antihypertensive Peptides

#### 4.2.1. AI-Enhanced Molecular Docking

Molecular docking is a bioinformatics-based computational simulation technique that focuses on predicting the interaction pattern and binding affinity between ligand and receptor molecules. By modelling the three-dimensional interactions between molecules, this technology provides an important theoretical basis for understanding the recognition mechanism of biomolecules and for drug design [82]. Molecular docking is widely used to analyze the action mechanisms of multi-target antihypertensive peptides. However, traditional molecular docking tools still face many challenges. These challenges include computational speed, binding mode accuracy, and binding free energy prediction accuracy. To solve these problems, researchers have developed AI-based molecular docking methods such as KarmaDock [83] and CarsiDock [84]. KarmaDock is a molecular docking method based on DL, which can quickly and accurately predict protein-ligand binding conformation and its binding strength. In terms of speed, KarmaDock takes only 0.017 s for a single docking, which is more than a hundred-fold faster than the traditional method. In terms of accuracy, KarmaDock is far better than traditional molecular docking tools. CarsiDock is also an innovative molecular docking method based on DL, and its model architecture is based on Transformer networks and references the design ideas of AlphaFold2. The method integrates a conformational rationalization module, which can effectively ensure the accuracy of ligand molecular atomic coordinate prediction. The above studies demonstrated that the molecular docking method based on AI significantly improved the accuracy and efficiency of docking. This technological advancement provides more reliable computational models and more accurate prediction results for target prediction and mechanism of action resolution of multi-target antihypertensive peptides.

#### 4.2.2. Network Pharmacology Analysis

Network pharmacology is an emerging interdisciplinary discipline that combines systems biology, pharmacology, bioinformatics, computer science, and complex network theory, with the aim of studying the interactions between drugs and organisms at the system level [85]. Network pharmacology elucidates the multi-target and multi-pathway mechanisms of drug action by establishing and analyzing the complex interrelationships among drugs, targets (e.g., proteins, genes), diseases, and biological pathways, thereby achieving the optimization of drug development and therapeutic strategies. The concept of network pharmacology was first proposed by Andrew L. Hopkins in 2007 [86]. With the deepening understanding of network pharmacology among researchers and the increasing recognition of its unique advantages, this methodology has emerged as a significant tool in the field of bioactive peptide research. By systematically analyzing the multi-target mechanisms of action between bioactive peptides and diseases, network pharmacology has significantly accelerated the study of multi-target antihypertensive peptides. Cao et al. [87] utilized network pharmacology and molecular docking technology to reveal that duck-derived bioactive peptides regulate blood pressure-related pathways by acting on the core targets of ACE, MMP2, REN, NOS3 and VCAM1, among which the key peptides, IPIIDYEVSITLGDPEALRDLLGEWVPWQ, are stably bound to the target proteins through hydrogen bonding, which provides a rationale for elucidating the mechanism of their antihypertensive activity. This provides a theoretical basis for elucidating its antihypertensive mechanism. Researchers have employed an AI and network pharmacology approach to identify peptides targeting multiple pathways for hypertension regulation. For instance, Bao et al. [88] screened novel multi-target antihypertensive peptides from highland barley proteins by integrating ML and network pharmacology approaches. The research team utilized a gradient boosting decision tree (GBDT) model to efficiently predict and validate a variety of potentially bioactive peptides, among which the peptide FPRPFL exhibited the strongest ACE inhibitory activity. Network pharmacology further indicated that the peptide synergistically exerted antihypertensive effects through multiple targets (e.g., ACE, AGTR1, AKT1) and multiple pathways. Collectively, these studies underscore the significant advantages of network pharmacology in predicting potential targets and constructing action networks of antihypertensive bioactive peptides. It is noteworthy that by integrating ML algorithms with the multidimensional data analysis technology of network pharmacology, researchers have initially developed an intelligent screening system for antihypertensive peptides. This system is based on the synergistic effect of multiple targets, providing a significant technical pathway for the development of innovative antihypertensive peptides characterized by network-regulated targets. However, network pharmacology is confronted with multiple challenges in its application, primarily manifested in the suboptimal data quality of databases and the limited capacity for identifying novel drug targets [89]. Crucially, targets predicted by network pharmacology methods are frequently associated with a risk of false positives. Consequently, potential targets initially identified by network pharmacology must undergo rigorous experimental validation to confirm their biological efficacy and pharmacological significance.

The research workflow for screening multi-target antihypertensive peptides based on network pharmacology can be systematically organized into the following steps. Initially, to predict potential targets of antihypertensive peptides using tools such as SwissTargetPrediction and Super-PRED. Then integrate the hypertension-related targets through disease databases, including CTD, GeneCards, and OMIM. To map the intersection between peptide-predicted targets and hypertension-associated targets via the Venny online tool to identify core candidate targets. Subsequently, construct a protein-protein interaction (PPI) network using STRING, and perform topological analysis with Cytoscape (version 3.10.3, Cytoscape Consortium, Seattle, WA, USA) to identify key nodes. Conducting the Gene Ontology (GO) functional annotation and Kyoto Encyclopedia of Genes and Genomes (KEGG) pathway enrichment analysis on the Metascape platform. This systematically elucidates biological pathways involved in vascular tone regulation, ion channel homeostasis, and the renin-angiotensin system, thereby establishing a multidimensional “peptide-target-pathway” interaction network. This framework provides theoretical support for clarifying the multi-target synergistic mechanisms of antihypertensive peptides. To facilitate network pharmacology research, Table 2 summarizes commonly used target prediction tools and public disease databases with their websites.

## 5. AI-Driven Screening for Antihypertensive Peptides: Challenges and Future Perspectives

### 5.1. Challenges in AI-Driven Screening of Multi-Target Antihypertensive Peptides

**Figure 3:**
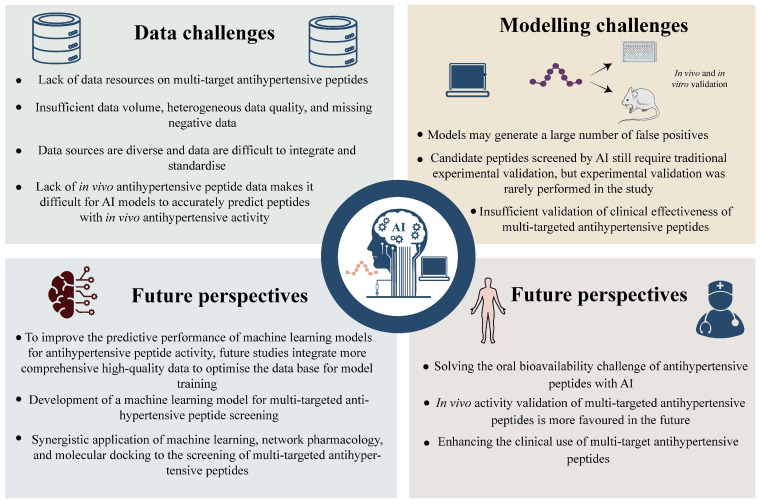
Challenges and prospects of AI for multi-target antihypertensive peptides.

Despite the utilization of AI in the screening of multi-target antihypertensive peptides, numerous challenges persist. Firstly, in terms of data, we face challenges such as insufficient data quality and scale. Data is the basis for AI model training, and its quality directly affects the predictive performance of the model. At present, although there are several active peptide databases (including a specialized antihypertensive peptides database), there are still problems such as insufficient data volume, uneven quality, and missing negative data, which seriously constrain the development of ML models. In addition, most of the antihypertensive peptides in the existing databases are only for the single target of ACE, while peptide data for other key targets (e.g., ACE2, renin, among others) are extremely limited. Since the screening of multi-target antihypertensive peptides requires the simultaneous acquisition of activity data for multiple targets, the expansion and optimization of the database still need to rely on a large number of experimental validations, and the high cost of acquiring high-quality experimental data further limits the training effectiveness of the model. Secondly, the complexity of the action mechanisms of multi-target antihypertensive peptides increases the difficulty of modelling. Multi-target antihypertensive peptides often involve multiple system pathway interactions, such as RAAS and the ETS. Consequently, constructing a multi-target antihypertensive peptide ML model is significantly more challenging than for a single-target peptide. The effective simulation and prediction of this complexity are a major challenge for AI modelling. Figure 3 summarizes current challenges, like data quality and in vivo validation, while also outlining future directions such as developing more precise predictive models, strengthening clinical research, and enhancing both in vitro and in vivo experimental validation.

**Figure 4:**
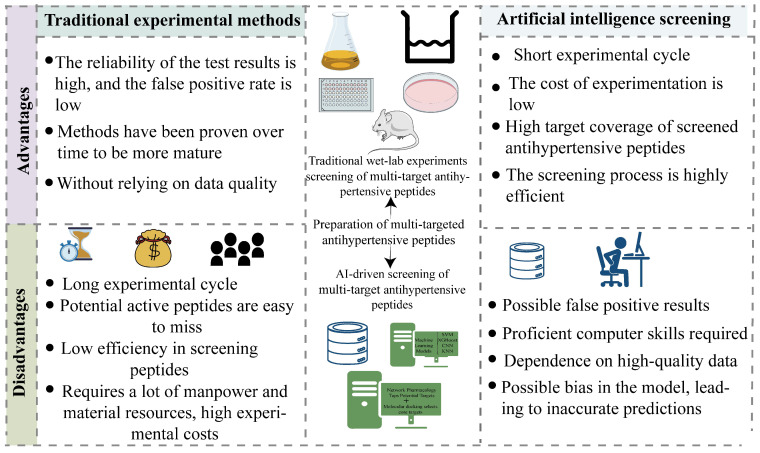
Advantages and disadvantages: Traditional vs. AI methods for multi-target antihypertensive peptides screening.

Antihypertensive peptides identified through AI prediction often exhibit discrepancies between in silico predictions and wet-lab experimental measurements. Therefore, the validation of multi-target antihypertensive peptides screened using an AI model is still required, both in vitro (e.g., measurement of ACE/renin inhibitory activity) and in vivo (e.g., assessment of blood pressure-lowering effect in an animal model), in order to avoid the problem of inconsistency between the predicted and the experimental results. First, if multi-target peptides need to be validated individually for each target activity, the experimental period is protracted, and the cost is elevated, which in turn increases the difficulty of the research. Second, discrepancies frequently emerge between in vitro and in vivo experimental results. The in vitro activity cannot reflect the actual in vivo bioavailability. Furthermore, in vitro experiments fail to truly simulate the complex physiological environment in vivo, such as inter-organ interactions (e.g., hepatic metabolism) and intercellular signaling, all of which contribute to the discrepancy between in vitro and in vivo activities [102]. The route of administration of antihypertensive peptides in animal experiments also significantly influences the interpretation of results. Intravenous injection and oral administration are two commonly used approaches for validating the activity of antihypertensive peptides in animal models. Intravenous injection ensures that the drug enters the circulation in its intact form, thereby enabling direct assessment of its maximal efficacy. Oral administration more closely approximates the real scenario in which humans ingest antihypertensive peptides through food and is simple to perform. However, oral administration can render peptides susceptible to degradation and metabolism during the processes of digestion and absorption, making it difficult for them to be absorbed intact into the systemic circulation [103]. This is also one of the reasons contributing to the discrepancy between in vitro activity and in vivo antihypertensive activity. Figure 4 presents a comparative analysis of the advantages and disadvantages between AI-driven and traditional experimental methods for screening multi-target antihypertensive peptides.

Achieving clinical application of antihypertensive peptides represents a highly promising research direction. Even though antihypertensive peptides have been successfully extracted from various food sources, clinical data lag far behind in vitro and in vivo experimental data, and this gap has become a core bottleneck restricting their clinical translation. Animal models play a vital role in the preliminary assessment of antihypertensive peptides, but significant discrepancies persist between animal model results and human clinical outcomes. This is largely attributable to species-specific differences and the fact that human hypertension is a multifactorial disease arising from the long-term, complex interplay of genetic, environmental, and other contributing factors, resulting in a pathophysiological mechanism considerably more intricate than that of existing animal models of hypertension [104]. Although these models can recapitulate certain features of hypertension, they fundamentally diverge from humans in terms of etiology, disease progression, and therapeutic responsiveness. Consequently, relying solely on in vitro or animal experiments to evaluate the antihypertensive potential of antihypertensive peptides is subject to considerable limitations.

### 5.2. Future Perspectives of AI-Driven Multi-Target Antihypertensive Peptides Screening

AI has been widely used in active peptide screening. Compared with traditional experimental methods, AI-assisted hypotensive peptide screening can save time and reduce costs. In view of the advantages of multi-target antihypertensive peptides in terms of their antihypertensive effects, future research trends should favor the use of AI for multi-target antihypertensive peptide screening. Establishing a database with more comprehensive and higher-quality data is imperative. This foundation is then to be built upon through the development of ML models and prediction tools applicable to multi-target antihypertensive peptides through innovative model training (Figure 4). It is recommended that future research endeavors focus on the further integration of ML with network pharmacology and molecular docking. In view of the fact that the current ML model is capable of predicting only peptide activity and is limited to single-target screening, a number of researchers have attempted to synergistically apply the aforementioned triple technology to the development of multi-target antihypertensive peptides. This represents an innovative concept that merits further exploration and expansion in subsequent research.

Bioavailability is a crucial parameter for evaluating the capacity of antihypertensive peptides to effectively enter the body and exert their pharmacological effects. Currently, most antihypertensive peptides are difficult to translate clinically into drugs due to their low oral bioavailability, and the majority are therefore used as dietary supplements. However, both for advancing the clinical application of antihypertensive peptides as pharmaceuticals and for enhancing their practical efficacy as dietary supplements, overcoming the issue of oral bioavailability is paramount. Furthermore, for humans, oral delivery is the most convenient and rapid method. Future research should focus on the development of non-injectable drug delivery systems, particularly the construction of delivery systems capable of enhancing both the stability and absorption efficiency of peptides. Future research should move beyond traditional enzyme inhibition assays and place greater emphasis on developing more physiologically relevant models that mimic the complex human physiological environment, so as to narrow the gap between in vitro antihypertensive activity and in vivo effects, as well as the differences observed between animal models and human clinical trial results. More importantly, the accumulation of more comprehensive clinical trial data is necessary to enable the clinical application of antihypertensive peptides. AI has great potential in the design of intelligent delivery systems, which can simulate peptide-nanomaterial interactions, predict encapsulation efficiency, release kinetics, and stability, thereby accelerating the screening of optimal carrier materials and formulations. Such an approach could lead to real improvements in the oral bioavailability of antihypertensive peptides.

## 6. Conclusions

This review summarizes the sources of multi-target antihypertensive peptides and elucidates the mechanisms through which they exert blood pressure-lowering effects via the synergistic regulation of the RAAS system and vascular endothelial function-related targets, and emphasizes the advantages of multi-target antihypertensive peptides in antihypertensive efficacy. On this basis, the review further discusses the driving role of AI in the screening of such peptides and summarizes the latest advances in related AI models. In particular, AI-coupled network pharmacology and molecular docking are highlighted as a novel strategy for the efficient discovery of multi-target antihypertensive peptides, which can significantly improve screening efficiency. This paper further outlines the overall workflow and commonly used tools involved in this strategy. Currently, the field still faces several challenges, including uneven data quality, the lack of dedicated AI models for multi-target antihypertensive peptides, inconsistencies between in vitro and in vivo antihypertensive activities, and difficulties in clinical translation. Future research should focus on constructing high-quality dedicated databases, developing accurate prediction models, and strengthening studies on bioavailability enhancement and clinical translation. In conclusion, AI is expected to accelerate the screening of multi-target antihypertensive peptides and promote the practical application of these natural bioactive peptides in functional foods and antihypertensive drugs.

## Figures and Tables

**Table 1:** Potential machine learning models for screening of antihypertensive peptides.

Bioactive Molecules	Name	Machine Learning Models Used	Model Performance	References
Peptide	pLM4ACE	Logistic regression (LR), RF, SVM, k-nearest neighboring (KNN), and multilayer perceptron (MLP)	pLM4ACE was a model trained based on logistic regression (BACC: 0.883, MCC: 0.77, AUC: 0.96) and constructed a webserver for antihypertensive peptide screening.	[12]
Peptide	AHTpin	SVM	AHTpin, an SVM-based predictive model for natriuretic peptides, showed high predictive accuracy and developed a web server for screening and predicting antihypertensive peptides.	[74]
Peptide	PAAP	RF	PAAP is an online web server based on RFalgorithm, capable of efficiently and accurately predicting peptide antihypertensive activity.	[75]
Peptide	mAHTPred	Adaboost, extremely randomized tree (ERT), Gradient boosting (GB), k-nearest neighbor, RF and SVM	mAHTPred is a model with good buck-peptide prediction performance (MCC: 0.767, AUC: 0.951) through multi-feature fusion, meta-learning frameworks and integration strategies and constructed a webserver for antihypertensive peptide screening.	[76]
Peptide	AHPP	Regression Decision Tree	AHPP achieves high accuracy (PLCC = 0.9513) prediction of hypotensive peptides by regression decision tree modelling and constructed a webserver for antihypertensive peptide screening.	[77]
Peptide	NA	CNN, Gated Recurrent Unit (GRU)	The research proposes a CNN-GRU hybrid deep learning model with multi-feature integration for predicting antihypertensive peptides, showing excellent prediction capabilities (ACC 99.10%, auROC 99.32%) constructed a webserver for antihypertensive peptide screening.	[78]
Peptide	Ensemble-AHTPpred	SVM, RF and XGBoost	The ML model Ensemble-AHTPpred achieved an overall accuracy of more than 90% on independent test data to accurately predict antihypertensive peptides.	[79]
Peptide	DeepForest-HTP	Deep Forest	DeepForest-HTP achieves an accuracy of 0.960, a sensitivity of 0.932, a precision of 0.989, and an AUC of 0.989 in the benchmark test.	[80]
Peptide	NA	XGBoost	The XGBoost model demonstrated an accuracy of 86.50% and an AUC of 94.11%.	[16]
Peptide	NA	Long short term memory (LSTM), BERT_base, ProtBERT, convolutional recurrent neural network (CRNN)	ProiBERT achieves current optimal performance in antihypertensive peptide screening (AUC nearly 0.9785).	[17]
Peptide	NA	CNN, SVM	The model achieves 95.0% accuracy on the benchmark dataset and 88.9% accuracy on the independent test set.	[81]

Note: BACC: Balanced accuracy; MCC: Matthews correlation coefficient; AUC: Area under the curve; PLCC: Pearson’s linear correlation coefficient.

**Table 2:** Common tools and functions for multi-target antihypertensive peptides studies in network pharmacology.

Name	Function	Web Address	References
SwissTargetPrediction	Predicting the target of an active ingredient’s potential action	http://swisstargetprediction.ch	[90]
Super-PRED	Predicting the target of an active ingredient’s potential action	https://prediction.charite.de/index.php	[91]
TargetNet	Predicting the target of an active ingredient’s potential action	http://targetnet.scbdd.com/home/index/	[92]
Pharm Mapper	Predicting the target of an active ingredient’s potential action	http://www.lilab-ecust.cn/pharmmapper/index.php	[93]
Comparative Toxicogenomics Database (CTD)	Provides targets associated with hypertension	https://ctdbase.org/	[94]
GeneCard	Provides targets associated with hypertension	https://www.genecards.org/	[95]
OMIM	Provides targets associated with hypertension	https://mirror.omim.org/	[96]
DisGeNET	Provides targets associated with hypertension	https://www.disgenet.org/	[97]
PharmGKB	Provides targets associated with hypertension	https://www.pharmgkb.org/	[98]
Therapeutic Target Database (TTD)	Provides targets associated with hypertension	https://idrblab.org/ttd/	[99]
STRING	Constructing protein interaction networks	https://string-db.org/	[100]
Metascape	For GO function and KEGG pathway enrichment analysis	https://Metascape.org/	[101]

Note: All websites were accessed on 20 April 2026.

## Data Availability

No new data were created or analyzed in this study.
